# Trapping intermediates in metal transfer reactions of the CusCBAF export pump of *Escherichia coli*

**DOI:** 10.1038/s42003-018-0181-9

**Published:** 2018-11-14

**Authors:** Kelly N. Chacón, Jonathan Perkins, Zachary Mathe, Katherine Alwan, Ethan N. Ho, Melek N. Ucisik, Kenneth M. Merz, Ninian J. Blackburn

**Affiliations:** 1 Department of Chemistry and Biochemistry, Reed College, Portland, OR 97202 USA; 2 Department of Biochemistry and Molecular Biology, School of Medicine, Oregon Health & Science University, Portland, OR 97239 USA; 3 Center for Drug Discovery, Baylor College of Medicine, Houston, TX 77030 USA; 4 Department of Chemistry, Michigan State University, East Lansing, MI 48824 USA

## Abstract

*Escherichia coli* CusCBAF represents an important class of bacterial efflux pump exhibiting selectivity towards Cu(I) and Ag(I). The complex is comprised of three proteins: the CusA transmembrane pump, the CusB soluble adaptor protein, and the CusC outer-membrane pore, and additionally requires the periplasmic metallochaperone CusF. Here we used spectroscopic and kinetic tools to probe the mechanism of copper transfer between CusF and CusB using selenomethionine labeling of the metal-binding Met residues coupled to RFQ-XAS at the Se and Cu edges. The results indicate fast formation of a protein−protein complex followed by slower intra-complex metal transfer. An intermediate coordinated by ligands from each protein forms in 100 ms. Stopped-flow fluorescence of the capping CusF-W44 tryptophan that is quenched by metal transfer also supports this mechanism. The rate constants validate a process in which shared-ligand complex formation assists protein association, providing a driving force that raises the rate into the diffusion-limited regime.

## Abstract (web summary)

Kelly Chacón et al. report insights into the mechanism of copper transfer between CusF and CusB, which are involved in heavy metal export as a part of bacterial host-defense strategies. Their results indicate fast formation of a complex involving a shared metallosite with ligands derived from each partner, followed by slower intra-complex metal transfer.

## Introduction

Recent studies are revealing how mammalian hosts are able to co-opt copper toxicity as a defense against invading pathogens, and conversely how the latter combat host-defense strategies via highly specific multiprotein export pumps^1^. The CusCBAF complex, unique to gram-negative bacteria, is one such system that confers pathogenic virulence and belongs to the resistance-nodulation-division (RND) family of heavy metal exporters^2–6^. The *cus* operon encodes four structural genes, denoted as *CBAF*, where CusCBA forms a tripartite complex which spans the periplasmic space, and imparts resistance to both Ag^+^ and Cu^+^^7–9^. CusA is a homotrimer (MW 115 kD, 1047 amino acids (aa)) with 12 transmembrane helices per monomer and two periplasmic domains, one of which contains the Ag(I)/Cu(I)-binding (Met)_3_ ligand set. The trimer binds six molecules of the adaptor protein CusB (44 kD, 407 aa)^10,11^, whose N-terminal metal binding site (M21M36M38) predicted from copper resistance of mutated *cusB* strains in a *ΔcueO* background^8^ is disordered in the crystal structures. The outer membrane protein CusC (51 kD, 460 aa) completes the envelope-spanning tripartite complex interacting with the BA complex so as to form a funnel-shaped contact^12^ allowing the transported metal to enter the 25 Å diameter pore and thus exit into the extracellular space. The final gene encodes a small soluble periplasmic protein CusF (12.2 kD, 110 aa), which has no homologs in the related RND multidrug-resistant exporters^13^, suggesting a role as a periplasmic copper chaperone.

The metallosites of the Cus system components have rich coordination chemistry that is dominated by methionine ligation. CusF coordinates a single Cu(I) or Ag(I) ion in a (Met)_2_His environment capped by a unique π−cation interaction with a tryptophan residue^14–16^. The ligand set provides a favorable site for Cu(I) coordination although the role of this Tryp residue is not fully understood. Functional suggestions include protection of the Cu(I) from O_2_ oxidation and/or increase in selectivity for univalent cations such as Cu(I) and Ag(I), although a role in assisting metal transfer reactivity is also possible. CusB contains three conserved Met residues in its disordered N-terminal domain which have been implicated from our own studies as the binding site for Cu(I) and Ag(I) by XAS^17–19^. DFT and QMMM calculations have provided further insight through in silico structures of the N-terminus in both apo- and metal-bound forms^20^. Metal binding appears to induce a moderate structural rearrangement suggestive of a function involving a metal-induced conformational switch. The CusA metal binding determined from crystal soaks lies within a deep cleft in the periplasmic domain and has been suggested to be comprised of three Met residues, although other potential ligands (particularly E625) are within coordinating distance^21,22^.

Structure/function relationships in copper transporters have typically been explored by determining minimal inhibitory metal concentrations on cell growth (MIC values) or by affinity tagging of one component, followed by separation and analysis of the extent of transfer^23–25^. Nuclear magnetic resonance (NMR) approaches^26^ have also proved useful in generating a consensus mechanism in which donor acceptor pairs interact across a specific protein−protein interface and share metal ligands in the transfer intermediate. For the CusF−CusB interaction a ligand-sharing intermediate has been proposed using in silico modeling^27^. However, while these studies have illuminated the residues involved in transport pathways, they provide little information on the efficiency of the process since direct measurement of rate constants has been frustrated by the similarity of the coordination chemistry of donor and acceptor sites of metal transfer pairs, and the consequent lack of unique spectral features for kinetic detection. Compounding the issue, transfer complexes are usually in the Cu(I) state which is invisible to many spectroscopic techniques. We have developed an alternative approach via the use of selenocysteine (SeC)^28,29^ or selenomethionine (SeM)^17,30^ labeling of the Cu(I)-coordinating Cys or Met ligands in one member of the donor−acceptor pair, where the unique Se-Cu feature in the Se K-edge extended X-ray absorption fine structure (EXAFS) spectrum is used to follow metal transfer into or out of the Se environment. The utility of the method has already been proven in a number of reports leading to proposals for the individual roles of the protein components of the *cus* transporter^18,30,31^. These studies have established that CusF is able to sense the periplasmic metal load, and under high flux transfers Cu(I)/Ag(I) to CusB, generating an active CusB conformer which binds to CusA and activates the pump. Single-molecule fluorescence resonance energy transfer (FRET) studies have recently proposed that the activation mechanism may involve shifting the equilibrium from partially assembled to fully assembled (envelope-spanning) forms of the pump^32^. The activated form can now also accept metal from the CusF chaperone, and transport it out of the cell. However, as the periplasmic metal flux falls, back-transfer from B to F leads to the apo-form of CusB which can no longer interact with CusA and shuts off further transport^31^.

Here we extend these studies to determine the mechanism of the reversible transfer of Cu(I) from CusF to CusB using selenomethionine labeling. In what we believe to be a pioneering study, we have interrogated the time course of the reaction by rapid freeze quench (RFQ) generation of samples coupled to measurement of the extent of transfer from the intensity of the Se−Cu interaction in the Se K absorption spectrum. Comparison of these data to the rate of fluorescent quenching of the CusF metallo-tryptophan interaction^33^ allowed us to formulate a multistep mechanism in which the CusF and CusB form an initial complex with shared ligands followed by much slower transfer chemistry. Finally, parallel studies at the Cu K edge of samples prepared at low temperature (4 °C) revealed the identity of the shared ligands in the intermediate as two methionines from CusB and one Met from CusF.

## Results

### Selenomethionine complexes of CusF and CusB

In previous work we demonstrated the utility of selenomethionine labeling of Cu(I)-Met transfer sites to track metal ion movement from a Se-labeled donor to a nonlabeled acceptor (or vice versa)^31^. This method relies on monitoring the intensity of the Se−Cu peak in the Fourier transform of the Se K EXAFS spectrum, as the copper transfers out of the Se environment of the donor into the S environment of the acceptor protein, and as documented below can be used to determine the metal status of each member of the donor−acceptor pair in real time during active transport. To maximize the efficiency of the method, all Met residues in the labeled protein should be ligands to copper, since uncoordinated SeM residues will contribute to the total Se signal (Se-C) yet have no Se−Cu peak in the transform. The constructs used in this study were designed to meet this requirement.

**Fig. 1:**
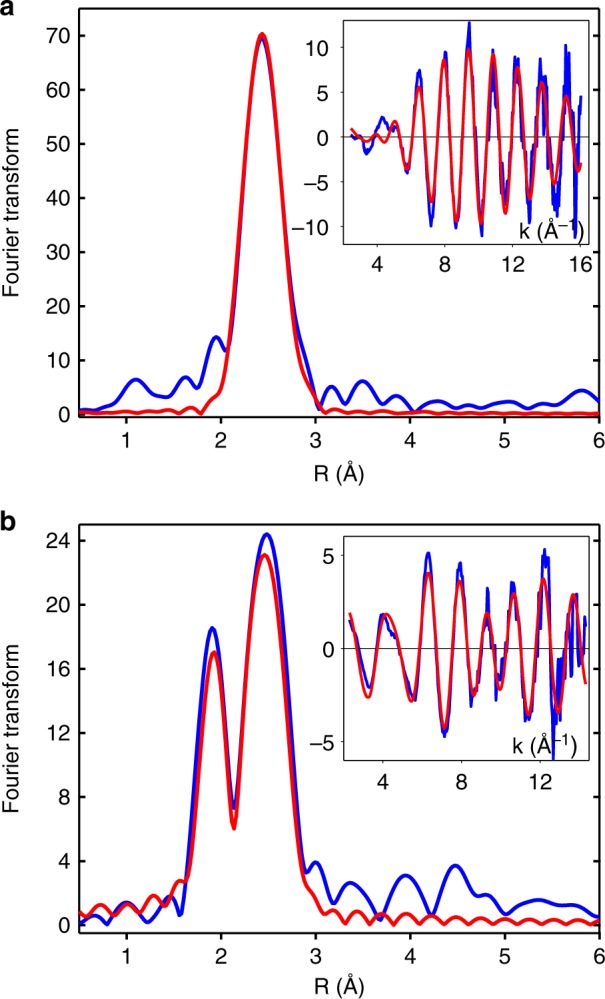
Cu and Se K EXAFS and Fourier transforms of SeM-labeled CusB-NT fully loaded with Cu(I). **a** Cu data. **b** Se data. Blue traces represent experimental data and red traces are simulations. Parameters used to generate the simulated spectra are listed in Table 1

The reaction between CusF and CusB can be run in either direction, starting from Cu(I)-loaded CusF and apo-CusB, or from apo-CusF and Cu(I)-loaded CusB. Since both proteins contain Met residues at their Cu(I) binding site, the SeM label can be incorporated on either protein. Therefore we needed protein constructs for each protein in which all Met residues were Cu(I) ligands. For CusB, the N-terminal fragment residues 1–61 shown previously to be competent for CusCBA activation^19,31^ fulfills the requirement with M21, M36 and M38 as metal-binding ligands^19,20^. For CusF the metal-binding M47 and M49 are accompanied by two additional noncoordinating Met residues M8 and M59. These latter residues were mutated to isoleucine to create the double mutant M8M59I and all CusF experiments described in this paper used this double Met mutant (hereafter referred to as CusF). Figure 1 shows the Cu and Se EXAFS and Fourier transforms of fully loaded CusB-NT. Supplementary Figure 1 shows similar data for wild-type CusF and the CusF double Met mutant, where the doubling of the intensity of the Se−Cu peak in the Se K transform of the latter shows the effect of mutating the noncoordinated SeM residues. Table 1 contains simulation parameters for these fully loaded (1:1) complexes that calibrate the Se−Cu bond-lengths and Debye−Waller factors for use in subsequent real-time transfer experiments.

**Table 1:** Fits obtained to the Cu K-EXAFS CusF and CusB SeM derivatives by curve-fitting using the program EXCURVE 9.2

	*F* ^a^	No^b^	*R* (Å)^c^	DW (Å^2^)	No^b^	*R* (Å)^c^	DW (Å^2^)	No^b^	*R* (Å)^c^	DW (Å^2^)	*E*_0_
Copper edge		Cu-N(His)^d^			Cu-S			Cu-Se			
WT CusF M8IM59I	3.08	1	2.00	0.003				2	2.38	0.007	−3.3
WT CusB	0.93							3	2.41	0.007	−5.0
Intermediate SeM-CusF	1.49	1	1.98	0.009	1	2.26	0.011	1	2.43	0.009	−2.9
Intermediate SeM-CusB	2.3				1	2.25	0.010	2	2.43	0.009	−3.0
Se edge		Se-C						Se-Cu			
WT CusF M8IM59I	0.47	2	1.97	0.005				1	2.38	0.005	−6.6
WT CusB	0.83	2	1.96	0.004				1	2.42	0.005	−5.5
Intermediate SeM-CusF	1.78	2	1.97	0.007				0.6	2.42	0.006	−6.3
Intermediate SeM-CusB	1.51	2	1.96	0.004				0.53	2.44	0.009	−4.9

*WT* wild type

^a^*F* is a least-squares fitting parameter defined as $F^2 = \frac{1}{N}\mathop {\sum}\nolimits_{i = 1}^N {k^6} ({\mathrm {Data - Model}})^2$

^b^Coordination numbers are generally considered accurate to ±25%

^c^In any one fit, the statistical error in bond-lengths is ±0.005 Å. However, when errors due to imperfect background subtraction, phase-shift calculations, and noise in the data are compounded, the actual error is probably closer to ±0.02 Å

^d^Fits included both single and multiple scattering contributions from the imidazole ring

### Freeze quench kinetics of the CusF−CusB interaction at the Se K edge

**Fig. 2:**
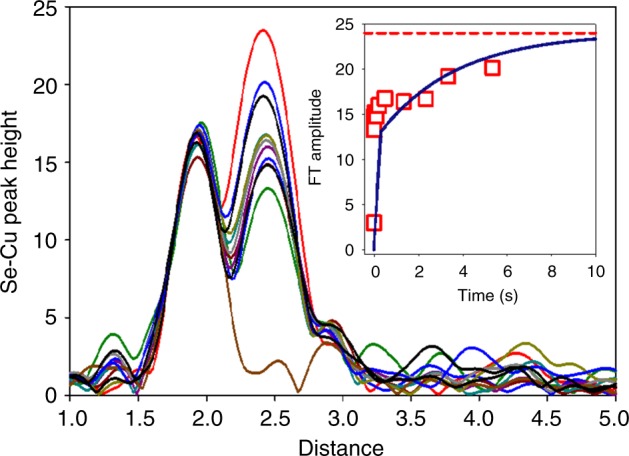
Time course of the transfer of copper from Cu(I)-loaded CusB-NT to SeM-labeled apo-CusF. The figure shows the increase in Se−Cu peak height in the Fourier transform of the Se K EXAFS as Cu(I) transfers to CusF and binds to the Se atom of the selenomethionine ligands. Traces from the bottom are for time points (in ms), 15, 46, 114, 216, 1340, 2340, 3340 with the final trace (red) representing the reaction end point at 30 s. The inset shows the Se−Cu peak height data plotted against time and fitted to a kinetic model involving initial rapid protein-protein complex formation (*k*_fast_ = 10^6^ M^−1^ s^−1^) followed by rate-limiting intramolecular metal transfer to form products (*k*_slow_ = 0.3 s^−1^). Simulated Se−Cu peak heights for each time point are listed in Supplementary Figure 1

As demonstrated in Fig. 1 and Supplementary Figure 1, the Se−Cu peaks in the Fourier transform of the SeM derivatives of CusF and CusB provide a unique spectroscopic signal of Cu(I) loading. We have used this signal to measure the kinetics of transfer from CusF to CusB (forward reaction), and from CusB to CusF (reverse reaction). Figure 2 shows the result of rapid mixing at 23 °C and freeze quenching of 1:2 mixtures of apo-SeM-CusF with Cu(I)-loaded S(met)-CusB-NT in 50 mM HEPES, pH 7.5 and 5% ethylene glycol, frozen into XAS cuvettes using a liquid ethane quench. The 1:2 protein ratio was specifically chosen to produce fully metalated SeM CusF at the end of the reaction. The figure plots Se−Cu Fourier transform amplitude versus time for time points collected from 15 ms to the end point at 30 s. The reaction is biphasic with a fast phase complete within 20–50 ms, and a slow phase occurring on the 500 ms to 10 s time scale. Particularly notable is the observation both from simple inspection and from simulation (Supplementary Table 1) that the amplitude of the fast and slow phase is approximately the same, each corresponding to the appearance of ~0.5 ± 0.1 Se−Cu interaction. The kinetic profile can be fit to a mechanism in which CusF and CusB react in a rapid step (*k* ~ 10^6^ M^−1^ s^−1^) to form a protein−protein complex with the Fourier transform amplitude indicating that the Cu atom is bound to one of the two SeM ligands in CusF, which subsequently rearranges (*k* = 0.3 s^−1^) to the final product—fully metalated CusF with copper bound to both of the SeM ligands:

$${\mathrm {Cu}}\left( {\mathrm I} \right){\mathrm {CusF}} + {\mathrm {apoCusBNT}}\mathop{\longleftrightarrow}\limits^{{k_{{\mathrm {fast}}}}}\left[ {{\mathrm {CusF}} - {\mathrm {Cu}}\left( {\mathrm I} \right) - {\mathrm {CusBNT}}} \right],$$

$$\left[ {{\mathrm {CusF}} - {\mathrm {Cu}}\left( {\mathrm I} \right) - {\mathrm {CusBNT}}} \right]\mathop{\longleftrightarrow}\limits^{{k_{{\mathrm {slow}}}}}{\mathrm {apoCusF}} + {\mathrm {Cu}}\left( {\mathrm I} \right){\mathrm {CusBNT}}.$$

The analysis implies that the first step generates an intermediate complex of CusF and CusB that contains a copper site with shared ligands since the copper starts off bound to 3 SMet ligands in the fully loaded CusB protein, yet has picked up one SeM ligand in the protein−protein complex. The data would be consistent with an intermediate comprised of one Met from CusF and two Mets from CusB.

**Fig. 3:**
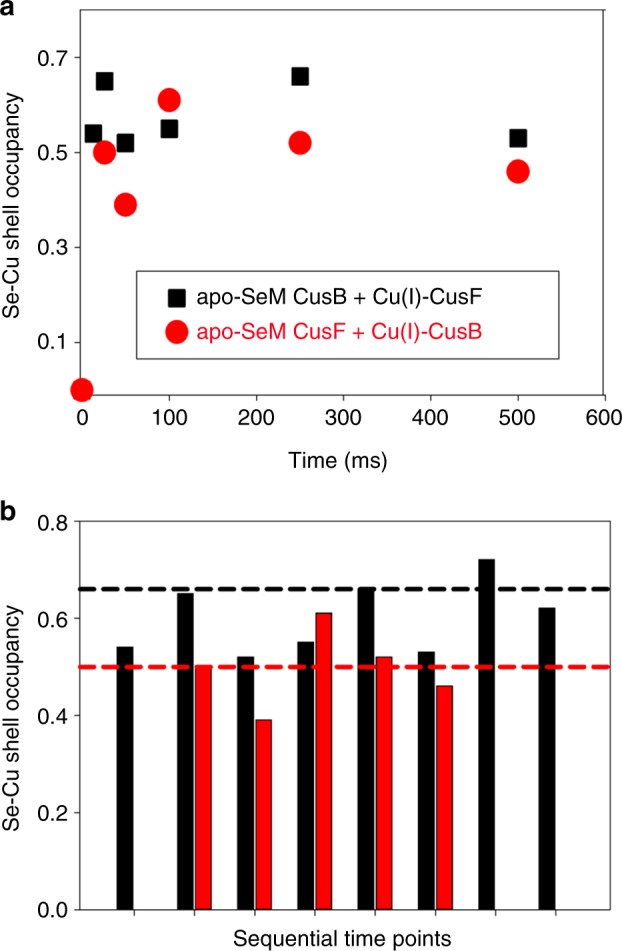
**a** Time course of the metal transfer reaction between CusF and Cu(I)-loaded CusB-NT at 4 °C. Red circles are simulated Se−Cu shell occupancies with the SeM label on CusF, black squares are simulated Se−Cu shell occupancies with the SeM label on CusB. In each case all Met ligands were selenium labeled. **b** Se−Cu shell occupancies plotted as a bar chart to emphasize the trends in value depending on whether the label is on CusF (red) or CusB (black). The red and black dotted horizontal reference lines are the values of Se−Cu shell occupancy predicted by the kinetic model for the shared ligand intermediate formed with the Se label either on CusF or CusB respectively. Sequential time points (from the left): 13 ms, 26 ms, 50 ms,100 ms, 250 ms, 500 ms, 1 s, 10 s. Simulated Se−Cu peak heights for each time point are listed in Supplementary Figure 1

To gain further insight we sought to slow the reaction by operating at 4 °C in the hopes of better resolving the initial fast phase, and stabilizing the putative intermediate for structural analysis. We carried out two sets of RFQ experiments, one set similar to the 23 °C data (apo-SeM-CusF + Cu(I)-S(Met)-CusB-NT) and one with the label switched to CusB (apo-SeM-CusB-NT + Cu(I)-S(Met)-CusF). The model predicts that if the shared-ligand intermediate can be stabilized, the Se−Cu intensity in the Fourier transform should simulate to 0.5 Se per Cu when the label is on CusF (one out of two SeM ligands bound to Cu) but increase to 0.67 when the label is switched to CusB (two out of three SeM ligands bound to Cu). Figure 3a, b shows the results of this analysis. The expected difference is close to the anticipated error of 25% in coordination numbers, but may still permit underlying trends to be observed. The data show two important results. First, while the initial phase is still too fast to resolve, the subsequent phase has slowed significantly, allowing the intermediate to fully form, since the Fourier transform amplitudes remain essentially constant between 26 ms and 1 s. Second, when the Se−Cu occupation numbers are compared, there is a trend towards higher occupation number when the label is on CusB, as predicted by the model. The data are tabulated in Supplementary Table 1, which gives average occupation numbers of 0.50 ± 0.08 and 0.6 ± 0.07 for Se-labeling of CusF and CusB respectively consistent with the expected trend for the model (see also Supplementary Figure 2).

**Fig. 4:**
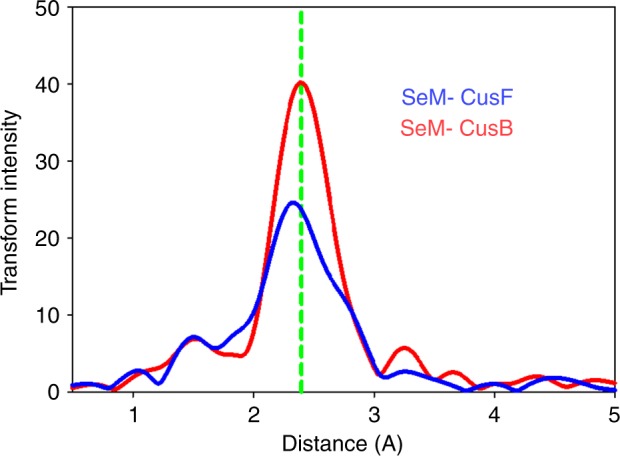
Fourier transforms of Cu K EXAFS of the intermediate formed at 500 ms in the metal transfer reaction between CusF and CusB. The red trace is the Fourier transform of data with the SeM label on CusB, while the blue trace is the Fourier transform of data with the SeM label on CusF. Experimental and simulated Fourier transform and EXAFS data corresponding to each situation is presented in Supplementary Figure 2 with parameters used in the fits listed in Table 1

Provided that the intermediate has built up to full occupancy as suggested by the near constancy of Se−Cu Fourier transform amplitude, Cu edge data can provide more accurate determination of the number of SeM ligands in the intermediate produced in each case. Figure 4 shows Cu edge Fourier transform data collected at 500 ms for SeM-labeled CusF (blue) and SeM-labeled CusB (red). Simple inspection of the Fourier transform amplitudes indicates large differences in copper coordination, while simulation tracks down these differences to the value of Cu−Se coordination number. When the label is on CusF, the Cu−Se shell occupancy is 1 SeM ligand per Cu(I). When the label is on CusB the Se−Cu occupancy rises to 2 SeM ligands per Cu(I) as predicted by the model.

The analysis reveals an additional complexity in the chemistry of the process since the identity of other ligands in the shared-ligand intermediate appears to depend on the location of the Se label. With the label on CusF, the best fit comprises 1 Cu−Se (CusF), 1 Cu−S (CusB) and 1 Cu−N(imidazole) from histidine (CusF). With the label on CusB the fit is more consistent with 1 Cu−S (CusF) and 2 Cu−Se (CusB). This suggests that a number of potential shared ligand states may exist either in equilibrium, or as sequential local minima on the reaction coordinate, and that the one that builds to full occupancy in the time course of our experiment is influenced by the nature of the starting material, i.e. the differential stability of Cu−S, Cu−N(imid) and Cu−Se bond strength.

### Static fluorescence spectroscopy of CusF

**Fig. 5:**
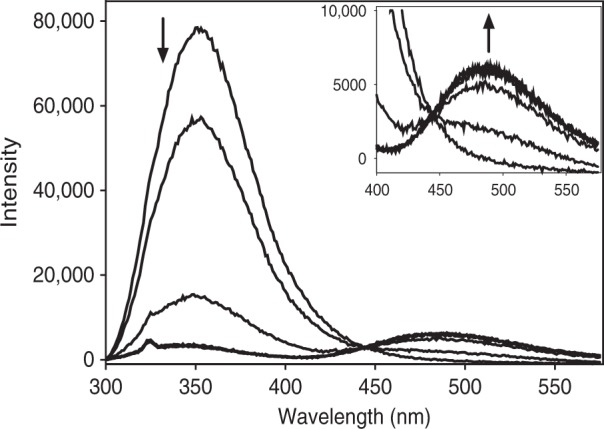
CusF W44 fluorescence quenching and formation of new 490 emission during titration with Cu(I). Increasing aliquots of Cu(II) were added to apo-CusF in the presence of 1 mM sodium ascorbate, in 50 mM HEPES pH 7.5. The main panel shows the decrease in 344 nm emission and the inset shows and expanded view of the concomitant increase in 490 emission (excitation wavelength 295 nm)

A unique feature of the CusF molecule is the presence of the π−cation interaction between W44 and the coordinated Cu(I)/Ag(I) ion. As reported by O’Halloran and coworkers^16^, the apo protein exhibits strong fluorescence from this tryptophan at 344 nm when excited at 295 nm, which is fully quenched by addition of Cu(I) at a 1:1 stoichiometry. Figure 5 and Supplementary Figure 3 show a similar titration of apo-CusF, conducted under strictly anaerobic conditions, using aliquots of an aqueous solution of Cu(II) sulfate slowly added to an anaerobic solution of apo-CusF in 50 mM HEPES pH 7.5 to which 1 mM ascorbate had been added. While [Cu(I)(CH_3_)_4_] in acetonitrile is generally a preferable reagent for conducting inorganic Cu(I) binding studies, we saw a marked undesired solvent quenching effect on overall fluorescence in our attempts to do such a titration, which is likely due to acetonitrile interacting with Trp 44. In either case, we observed complete quenching of the 344 nm emission of apo-CusF at a Cu(I) to protein ratio of 1:1. Interestingly, in our hands the titration shows that as the Cu(I) binds, a new luminescence signal grows in at ~490 nm which forms concomitant with the loss of the 344 nm emission, and exhibits an isosbestic point indicative of conversion of one species (apo-CusF) to another (Cu(I)-CusF). As a point of reference, luminescence at ~550 nm is observed in the CO complex of Cu(I) hemocyanin which emits at 540 nm when excited between 280 and 330 nm^34^ as well as the CO adducts of Cu(I) complexes of substituted imidazoles^35,36^. These emissions were assigned to decay of a triplet state which is populated by energy transfer from singlet states arising from the Cu(I)−CO interaction, most likely involving charge transfer from metal to empty π* levels on the CO ligand. It is likely that a similar photophysical mechanism may operate in the Cu(I)-W44 π-cation luminescence involving charge transfer between the filled d-shell and the π-antibonding levels of the tryptophan six-membered ring system.

To further test the premise that the ~490 nm emission is related to a Cu(I)-tryptophan π−acceptor interaction, we examined the luminescence properties of apo- and Cu(I)-loaded CusF-W44A. As shown in Supplementary Figure 3b, the variant showed no emission with or without added Cu(I). Additionally, an excitation scan of Cu(I)-loaded CusF, parked at ~490 nm, produces a sharp peak at ~296 nm, which corresponds to the canonical Trp excitation (Supplementary Figure 3c). These data provide confirmation that the 490 nm emission is both Cu(I) and W44 dependent, adding confidence in the assignment. As it was concerning that this feature did not appear in the earlier work of Xue and coworkers^16^, particularly at the high concentrations used there, we examined our experimental conditions carefully. We found that strict anaerobic conditions were a key requirement for the appearance of this ~490 nm feature, as shown in Supplementary Figure 3d. Even a brief bubbling of ambient air into an anaerobic cuvette of exhaustively dialyzed, anaerobic Cu(I) CusF was sufficient to completely quench the ~490 nm peak. As Cu(I) CusF is intrinsically quite stable in air, it is likely that the previous work may have inadvertently quenched this spectroscopic feature before its capture.

**Fig. 6:**
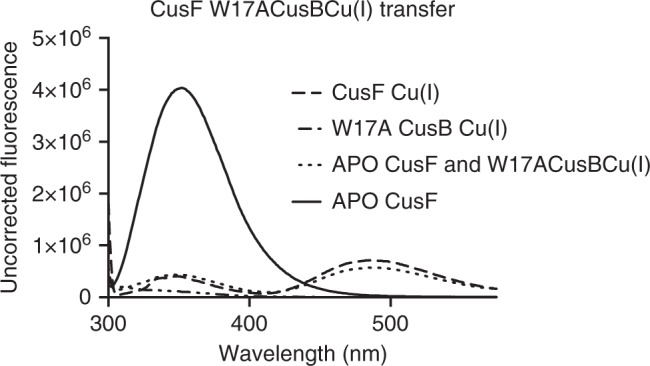
Transfer of Cu(I) from CusB to CusF monitored by concomitant decrease in 344 nm emission and increase in 490 nm emission

We utilized this ~490 nm feature as a means to confirm successful Cu(I) transfer from CusF to CusB, as it would conclusively link the quenching of the more intense ~350 nm peak to protein metallotransfer in the stopped-flow fluorescence experiments, which are limited to a single wavelength. Figure 6 shows fluorescence spectra of the incubation of a ratio of 1:2 apo-CusF and W17A Cu(I) CusB NT, the construct in which the nonessential Trp of CusB has been mutated in order to observe only the CusF fluorescence signal. The apo- and Cu(I)-loaded states of CusF are shown for comparison, and indicate that the Cu(I) was cleanly transferred from W17A CusB NT over to CusF, to an identical endpoint of the inorganic preparation of Cu(I) CusF. In this manner, the use of the ~490 nm signal allowed us to confidently assign the quenching at ~350 nm to the phenomenon of metallotransfer from CusB to CusF, and not to inorganic quenching. The ~490 nm signal was not tracked in the stopped-flow apparatus, as the low intensity of the band would require extremely high concentrations of protein in order to allow for good signal-to-noise of the fluorescence PMT detector. In contrast, the ~350 nm feature is of extremely high intensity and provided the needed signal to noise at fast timescales.

### Stopped-flow fluorescence kinetics of metal transfer

**Fig. 7:**
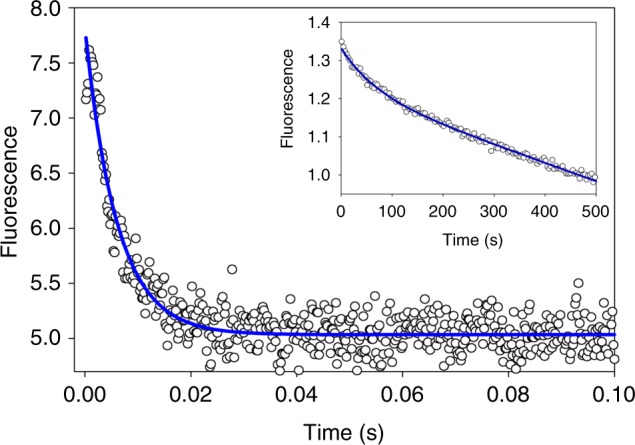
Stopped-flow fluorescence trace of the first 100 ms of reaction of unlabeled apo-CusF with unlabeled Cu(I)-loaded CusB-NT. Solid blue line is a first-order fit to the data with rate constant *k* = 165 s^−1^. The inset shows the subsequent 500 s of reaction fitted to an exponential decay (*k* = 0.012 s^−1^) with a linear component to account for photobleaching and/or other nonreaction-dependent photophysical processes

Copper binding to CusF is characterized by quenching of the 344 nm luminescence of the capping W44 ligand. Kinetic profiling of the W44 signal should therefore report on the dynamics of the W44 ligand during CusF−CusB interaction that is distinct yet complementary to RFQ-XAS data. We followed the rate of quenching of the 344 nm emission when apo- S(met)-CusF was mixed with Cu(I)-S(Met)-CusB-NT (i.e. both proteins unlabeled) in the stopped flow at 4 °C. As shown in Fig. 7, a rapid first-order quenching of the 344 nm emission is observed on a time scale of 20–50 ms followed by a slower rate on the order of tens of seconds (*k*_fast_ = 165 ± 7 s^−1^, *k*_slow_ = 0.012 s^−1^). These data are broadly consistent with the RFQ XAS data, although not strictly comparable since neither protein was Se-labeled. When the experiment was repeated using apo-SeM-CusF and Cu(I)-S(Met)-CusB-NT, a similar result was obtained (Supplementary Figure 4) with rate constants *k*_fast_ = 98 ± 5 s^−1^ and *k*_slow_ = 0.015 s^−1^. The fluorescence decay is strictly first order suggesting a unimolecular event in contrast to the kinetic model used to fit the RFQ data where a bimolecular protein−protein association was assumed to be the initial reaction. This may imply that a number of sequential steps are involved in CusF−CusB shared-ligand complex formation, the first of which may be protein−protein association, followed by W44 344 nm emission decay within this pre-equilibrium complex. In any event it appears likely that the decay in 344 nm emission accompanies the formation of the intermediate characterized by XAS, but the fluorescence is only partially quenched at this point, suggesting that W44 is not yet fully associated with the CusF metal-binding site. Full quenching of the W44 signal occurs on a longer time scale consistent with the resolution of the intermediate into final products.

The stopped-flow data have allowed us to compare the effect of Se-labeling on the kinetics of the decay process, and thus to obtain a crude assessment of its effect on the electronics of the reaction. Se-labeling decreases the rate of initial 344 nm emission decay from 165 to 98 s^−1^, a factor of 1.7, whereas the rate of subsequent reaction to products exhibits a modest increase from 0.012 to 0.015 s^−1^, a factor of 1.3 (Supplementary Figure 4). These data indicate that the overall kinetic profiles are maintained while the absolute values of the rates exhibit only modest differences between S and Se, thereby validating the labeling strategy. Notwithstanding, while it appears that the W44 emission decay kinetics are qualitatively consistent with the RFQ data, it has not yet been possible to obtain rate constants on comparable phases or with comparable conditions of temperature and labeling to make direct comparisons.

## Discussion

We have used selenomethionine labeling coupled to RFQ XAS at the Se and Cu edges to investigate the mechanism of interaction between the metallochaperone CusF and its cognate partner CusB. Our data indicate a multistep process involving the initial formation of one or more CusF−CusB intramolecular complexes that lead to an intermediate involving copper coordinated by ligands derived from both proteins. The rate of formation of the shared ligand complex is fast and occurs on a sub-millisecond to tens of milliseconds time scale, while the shared ligand complex decays to products more slowly (seconds) at room temperature, and is sufficiently stable to become fully populated at 4 °C such that its ligand set can be determined. EXAFS analysis of the intermediate at the Cu and Se edges suggests that the ligand set is dynamic and depends on whether the SeM label is placed on CusF or CusB. Whereas the coordination is always 3-coordinate, SeM labeling of CusF generates an intermediate involving two CusF residues (Met and His) and one CusB residue (Met), whereas labeling CusB generates a 3 Met species, one from CusF and two from CusB.

The concept of chaperone-target intermediates where the metal being transferred is shared between ligands of the donor and acceptor was first proposed by Rosenzweig and O’Halloran based on their observation of a shared ligand complex in the homodimeric crystal structure of ATOX1^37^. The driving force was suggested to arise from donor acceptor molecular recognition, allowing metal transfer to proceed down a shallow thermodynamic gradient involving rapid reversible metal exchange within 3-coordinate shared ligand complexes. Indirect experimental evidence for the 3-coordinate intermediate was subsequently obtained from NMR studies of the interaction of yeast Atx1 with an MBD1 of its cognate partner Ccc2 where the donor−acceptor complex detected between the wild-type proteins was disrupted in the absence of Cu(I) or when specific Cys residues were mutated to alanine^26^. The work provided evidence of a metal-mediated intermediate where one cysteine from each protein was an essential component. The intermediate species was in fast chemical exchange with the free proteins (>10^3^ s^−1^) suggestive of a rapid pre-equilibrium complex formation. Molecular mechanics calculations are largely in agreement with these conclusions, although they leave open the possibility that the transfer intermediate is 2-coordinate, sharing only a single Cys residue from each partner^38^.

Transfer intermediates have also been investigated using single-molecule FRET methodologies, where interactions between donor and acceptor are monitored by observing FRET signals between different fluorescent tags attached to each protein^39–41^. These studies confirm the formation of protein−protein complexes at the single-molecule level and allow the kinetics of their formation and decay to be estimated from the residence time of the FRET signal attributed to a specific protein−protein conformation. Interestingly, a number of different conformations are observed for the ATOX1-MBD4 and ATOX1-MBD34 respectively (where MBD34 represents the domain3-domain4 dimeric construct), and complexes of the chaperone with ATP7B where “face to face” and “face to back” conformers coexist. The ‘face to face” species represent the ligand-sharing intermediates proposed previously, yet curiously their existence is not strictly metal-dependent, although the population of this state appears to be enhanced in the presence of Cu(I). These studies suggest an extremely dynamic metal ion capture mechanism where multiple conformations of the donor−acceptor diad may exist, and possibly assist in the formation of the reactive 3-coordinate intermediate.

**Fig. 8:**
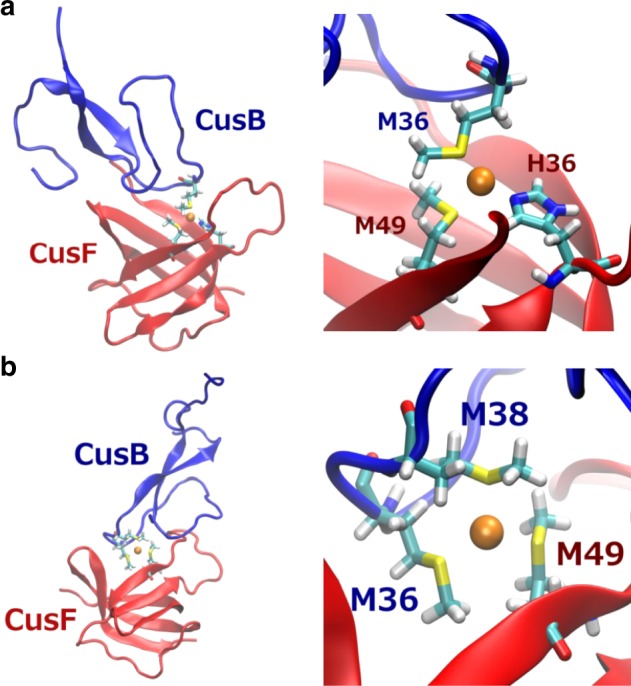
Models for the structure of the shared-ligand intermediates. **a** The MMM intermediate involving two Mets from CusB-NT and one Met from CusF. **b** The HMM model involving one Met from CusB-NT and a Met and a His from CusF. Structures on the left are in silico models of the protein−protein interactions between CusB-NT and CusF showing the protein interface while those on the right depict the proposed active site structures of the intermediates. The models are reproduced from coordinates determined from the QMMM studies reported in ref. ^27^

Our present data provide evidence for a shared ligand intermediate in the reversible copper transfer reaction between CusF and CusB. Such an intermediate was proposed previously from DFT and QMMM methods which were used to generate an in silico model of the CusF-CusB-NT protein−protein complex^27^. The most populated model derived from docking simulations (termed the MMM model) showed Cu(I) sharing two CusB ligands (M36, M38) and one CusF ligand (M49) in complete agreement with the structure observed here from EXAFS analysis of the intermediate formed using CusB selenomethionine labeling. A structure of the MMM model based on the in silico coordinates which reproduces our experimental finding is shown in Fig. 8a. Interestingly the second most populated state generated from the docking protocol (termed the MMH model) involved M36 of CusB and M49 and H36 of CusF consistent with the Cu K EXAFS-derived structure for the intermediate formed with the selenomethionine label on CusF (Fig. 8b). As pointed out by Merz and coworkers^27^, it is conceivable that both of these intermediate structures are utilized as the transfer proceeds along the reaction coordinate, and it is therefore not unreasonable that seleno-labeling could modulate their relative stabilities and drive the reaction towards the intermediate state that contains the most favorable configuration for its Cu−methionine interactions. We note that while the Se edge does not distinguish between the MMM and the MMH models with the label on CusF, the Se edge data fully corroborate the MMM model when the label is on CusB.

We also studied the transfer reaction by following the quenching of W44 fluorescence. Our data show that the W44 fluorescence decreases in a two-step process with a fast phase (*k* = 165 s^−1^) and a slow phase (*k* = 0.012 s^−1^) which implies at least three conformational states for the W44 residue—the starting fully luminescent state, an intermediate state of lower luminescent intensity, and the final fully quenched state corresponding to the Cu(I)-bound π−cation interaction. The in silico model of the protein−protein complex between CusF and CusB shows the W44 loop flipped backwards to form a stacking interaction with the aromatic ring of CusB-F35, which allows access of the CusB metal-binding loop to the CusF site^27^. More recent QMMM calculations on uncomplexed CusF have shown that W44 is capable of conformational switching between the crystallographically observed closed state and an open conformation with the W44 loop flipped aside^42^. Our data suggest that the intermediate semi-quenched state forms on the same time scale as the shared-ligand complex. We propose that the observed partial quenching is due to binding of Cu(I) to a configuration where W44 is flipped back as represented by the in silico structures of the open or CusB-bound CusF forms, while the subsequent slow phase represents the resolution of this intermediate state to the fully quenched, π−cation form of the metallated CusF.

The value of the proposed protein−protein association rate constant of 10^6^ mol^−1^ s^−1^ is notable. The consensus mechanism of protein−protein interaction (PPI) is the formation of an initial so-called encounter complex where the two proteins approach and make contact in a diffusion-driven process^43,44^, resulting in second-order rate constants that span a wide range between 10^1^ and 10^9^ mol^−1^ s^−1^. Rates in the lower regime are suggestive of conformational rate limitation, where the process is limited by how fast the two proteins explore the conformational landscape of surface complementarity. In the upper time regime, the rates are limited by diffusion, with the cross-over point ~10^5^ mol^−1^ s^−1^. These faster rates are believed to be induced by specific interactions that provide a strong driving force for selection of the optimal surface conformation, and often involve electrostatic complementarity.

Simulation of the 25 °C RFQ data for CusF−CusB interaction suggest a PPI rate constant with a lower limit of ~10^6^ mol^−1^s^−1^, implying a mechanism where additional driving force assists conformational sampling of the interacting surfaces, and we suggest that the favorable energetics of formation of the shared-ligand intermediate might provide this extra driving force. However, given that the shared-ligand intermediate has by necessity a subset of the donor atoms present in the reactant molecules, any thermodynamic stabilization must be small, and the increase in association rate may be due largely to localized orientation and/or conformational effects. For example, metal exchange requires the W44 cap in CusF to flip to its open conformation which in turn will allow the proposed stacking interaction with CusB Y35.

Notwithstanding the detailed mechanism and/or energetics of protein−protein association, the data are consistent with a rate at the low end of the diffusion-limited regime. CusF may interact with a number of different partners in the periplasm depending on which pathway of metal efflux is operative. Studies to date have identified interactions with CopA^25^, CusB^18,45^, and CusA^31^, while association with other periplasmic proteins such as CusS and/or CueO are probable. The partitioning of copper among these different pathways is determined by factors that include the metalation state of CusF, and by the differential rates of protein−protein association of CusF with its interactome. Rapid protein−protein complex formation may be particularly important when the periplasmic copper flux becomes high enough to be toxic, requiring a fast and selective response. Our proposed mechanism provides for such an eventuality, and begins to furnish the quantitative framework for unraveling the complexity of periplasmic copper trafficking.

Two final caveats are worthy of discussion: firstly, the relevance to physiological copper concentration, and second, the mechanistic consequence of the CusB N-terminal truncation. The concentrations of samples for the Cu and Se XAS experiments (250 μM) are of necessity higher than intracellular concentrations to obtain data of sufficient signal to noise. However, the fluorescence measurements are made on protein samples at 20 μM or below, which approaches the concentration of copper in cells under physiological conditions. The correspondence between the mechanism of transfer derived from the two methods then suggests that the XAS data are indeed relevant to the physiological situation. However, the relevance to physiology is better discussed in terms of MIC values (minimum inhibitory concentrations) or minimum copper concentration required to completely inhibit growth. The MIC value for the *ΔcueO* genotype is quoted by Franke et al.^8^ to be 3.25 mM CuCl_2_. This suggests that the Cus export pump is overwhelmed at this concentration, but would be fully operative at copper concentrations 10−100-fold lower. Thus we can estimate that an extracellular concentration of 30–300 μM would lead to a functional pump. These estimates span the concentration range of the fluorescence and XAS experiments, and add confidence that the mechanistic insights are relevant.

With respect to the second caveat, the present study of necessity used the SeM-labeled CusB-NT truncate since the multiple noncoordinating Met residues of the full-length protein would render the Se signal insensitive to metal binding. We showed previously that the CusB-NT was both necessary and sufficient to impart copper resistance to a CusB deletion mutant of *E. coli* constructed in the *ΔcueO* background, albeit with reduced efficiency compared to wild type^19^. While this suggests that the mechanism of transfer from CusF is similar between N-terminal truncate and full-length CusB, we cannot exclude the possibility that elements of the transfer process may differ in the full-length CusB−CusF complex. For example, recent studies have shown that the full-length CusB is a dimer in solution, and undergoes a conformational change to a more compact state on Cu(I) binding^46,47^. As a consequence, metal-induced changes in the overall protein scaffold may complicate the simple reversible transfer behavior observed between CusF and CusB-NT, or even render it irreversible, as recently suggested by Meir and coworkers^46^ as a means of ensuring unidirectional Cu(I) efflux through the CusCBA complex. With the mechanism of reversible transfer between metallochaperone and CusB-NT now firmly established, comparison with the full-length protein should be informative, using only Se labeling of CusF, and we plan to pursue such experiments in the future.

## Methods

### Preparation of the double methionine to isoleucine mutant CusF

Site-directed mutagenesis was performed on CusF(6–88) to alter methionine residues M8 and M59 to isoleucine. CusF was expressed as a fusion with thioredoxin (trx) with a His_6_ tag and TeV protease cleavage site inserted between the N-terminal TrX and C-terminal CusF (residues 6–88) sequences (*CusF*_*(6−88*_*)trx-his6-tev)*. The pETDuet-1 plasmid containing the *CusF*_*(6-88*_*)trx-his6-tev* gene was purified from *E. coli* BL21(λ DE3). The *CusF*_*(6−88*_*)trx-his6-tev* PCR template was isolated via a *Nco*I/*Hin*dIII (NEB) digest and purified using the QIAquick PCR purification kit (Qiagen). Point mutations were introduced by the generation of two PCR products with overlapping end regions which were then purified and joined via a second round of PCR. Primer pairs were created so that the flanking primers of the original fragments could be used to join the two fragments together in the final PCR amplification. Amplification of both plasmid and gene was achieved using Deep Vent DNA Polymerase (NEB). Both the plasmid and the gene insert were then digested using *Nco*I and *Hin*dIII and ligated using T4 ligase (NEB) and transformed into *E. coli* BL21(λ DE3). Primer sequences are shown in Table 2.

**Table 2:** Primers used in this study

Primer name	Sequence
CusF M8I-F	5′ GTATTTTCAGGGCGAAACCATCAGCGAAGCACAACCAC 3′
CusF M8I-R	5′ GTGGTTGTGCTTCGCTGATGGTTTCGCCCTGAAAATAC 3′
CusF M59-F	5′ CACCCCGCAGACGAAAATCAGTGAAATTAAAACCGG 3′
CusF M59I-R	5′ CCGGTTTTAATTTCACTGATTTTCGTCTGCGGGGTG 3′
TrxAH6NCO-F	5′ AATAAACCATGGCCGATAAAATTATTCACCTGACTG 3′
CusF *Hin*dIII-R	5′ ATAATAAAGCTTTTACTGGCTGACTTTAATATCCTG 3′

### Expression and purification of S-Met CusF and S-Met CusB-NT

*E. coli* BL21 (λDE3) transformed with the Met to Ile double mutant *CusF*_*(6−88*_*)-trx-his6-tev* plasmid, and the W44A (Met to Ile double mutant) were obtained by incubation of a freshly streaked plate into LB media containing 100 μg/mL ampicillin at 37 °C until an OD_600_ of 0.8, then induced to produce protein with 0.4 mM of isopropyl β-d-1-thiogalactopyranoside and shaken at 37 °C for 4 h. Protein purification was carried out as described previously^31^. Cells were harvested by centrifugation, and lysed using the French pressure method, followed by further centrifugation to remove cell debris. The filtered supernatant was applied to an Ni-NTA column, rinsed once with buffer, and eluted with 250 mM imidazole. Tobacco etch virus (TeV) protease and 5 mM β-mercaptoethanol were added to the protein solution and the mixture was incubated at 20 °C overnight to remove the His6-Trx tag. After dialysis the protein solution was re-chromatogrammed on Ni-NTA to yield cleaved, pure apo-CusF. The protein was pure as judged by SDS-PAGE with the correct molecular weight (~10 kDa), and no visible impurities.

*E. coli* BL21 (λDE3) transformed with the CusB NT *trx-his6-tev* plasmid was grown and processed in the same manner as for CusF. The final product was pure as judged by SDS-PAGE with the correct molecular weight (~7 kDa), and no visible impurities.

The purified proteins were dialyzed against HEPES buffer (50 mM, pH 7.5) for subsequent metallation.

### Expression and purification of Se-Met CusF and Se-Met CusB-NT

The selenomethionine labeled variants of each of the aforementioned constructs were prepared as described previously^31^. Procedures were similar to production of S(Met) derivatives except that Met auxotrophic BL21(DE3) cells transformed with the CusF and CusB plasmids were grown in minimal medium that was supplemented with selenomethionine. The Se-Met variants of CusF and CusB-NT were purified and stored as described above, and their Se-to-protein content was verified by ICP-OES and the BCA assay.

### Metal reconstitution of Cus proteins

Cu(I) forms of S/Se-Met CusF and S/Se-Met CusB-NT were prepared by slow addition of 0.1 volumes of a stock solution of [Cu(CH_3_CN)_4_]PF_6_ in pure acetonitrile (ACN) to a 0.9 volumes of a solution of the apo-proteins in buffer, followed by serial dialysis against 10, 5, and 0% acetonitrile to remove excess Cu(I), and ACN as previously described^31^. The serial dialysis ensured that residual Cu(I) remained coordinated by ACN and was always protected from disproportionation in aqueous media. Metal to protein stoichiometries were verified by ICP-OES and the BCA or Bradford assay.

### Static fluorescence spectroscopy

All static fluorescence experiments were carried out using new thin-walled fluorescence cuvettes fitted with a screw-top septa (Starna, Inc.) using a Fluoromax-4 fluorescence spectrometer (Horiba Scientific). The instrument was calibrated by measurement of the Raman scattering of water, and cuvettes were monitored by water and buffer scans to reveal any evidence of protein or aliphatic contamination. All data were collected as corrected S1/R1 scans. For a typical experiment, freshly metallated proteins (when appropriate) were buffer exchanged into 50 mM HEPES, and all work was carried out at 4 °C using an in-instrument chiller. Protein concentration was kept to 5–10 μM for experiments involving only CusF or CusB, and for the purposes of this work, the CusF to CusB protein stoichiometry was kept at 1:2 during metal transfer experiments to insure full CusF W44 fluorescence signal development. In experiments that tested metal transfer from CusB to CusF, the W17A CusB NT construct was used to ensure that all fluorescence signal came only from W44 CusF. All work was carried out anaerobically. In the inorganic titration of Cu(I) and wild-type CusF, ascorbate was added to 1 mM to ensure full anaerobicity and thus maximize the fluorescence signal at 490 nm. All spectra were blank subtracted using either a 50 mM HEPES buffer scan, or a 50 mM HEPES buffer, 1 mM ascorbate scan when appropriate.

### Stopped-flow fluorescence spectroscopy

All stopped-flow fluorescence work was carried out using an SX-20 Stopped-Flow apparatus (Applied Photophysics, UK) configured for single wavelength fluorescence detection at ~350 nm using a 295 nm LED light source set to 2 amps (Applied Photophysics, UK) and a fluorescence PMT detector tuned to ~350 nm via an inline monochromator to avoid the use of filters to reject contaminating wavelengths from the photoemission. In order to capture an effective signal range and avoid photobleaching, the LED source voltage versus detector signal was calibrated for each experiment by mixing a given concentration (50–100 μM) apo wild-type CusF in 50 mM HEPES (the fluorescent protein of interest) against 50 mM HEPES buffer. Upon reaching peak Trp fluorescence signal, the high voltage (HV) of the detector was set to approximately 8 V in order to maintain sensitivity at lower fluorescence signals as the W17A CusB Cu(I) to wild-type CusF metal-transfer reactions progressed. In a typical stopped-flow experiment, ~50 μM anaerobic apo S/Se-Met wild-type CusF was mixed against ~100 μM S/Se-Met Cu(I) CusB NT and the reaction monitored from the millisecond range to the second range, depending upon the reaction phase under study. Scanning fluorescence was carried out in the stopped flow across the 300–550 nm range to obtain spectra of each reaction before and after mixing experiments. Blank controls were conducted by ensuring that 50 mM HEPES buffer produced no fluorescence by mixing buffer against itself under identical timeframes as that of protein experiments. Kinetic traces were simulated by nonlinear regression using Sigma Plot 12.0. The first 100 ms of fluorescent decay was simulated by the equation

$$F_t = F_0 + A{\mathrm {exp}}( - k_{{\mathrm {fast}}}t),$$

where *F*_0_ is the measured fluorescence at time *t*, *F*_0_ is the initial fluorescence, *A* is the amplitude of the fast phase, and *k*_fast_ is its rate constant. Subsequent decay was simulated by the equation

$$F_t = F_0 + B{\mathrm {exp}}\left( { - k_{{\mathrm {slow}}}t} \right) + Ct,$$

where *k*_slow_ is the rate constant for the slow phase, and other terms are defined as above but with the inclusion of a linear term which accounts for fluorescent decay via nonreaction-dependent photophysical processes, particularly photobleaching.

### Rapid freeze quench experiments

All RFQ experiments were carried out on a Quench Flow-3 apparatus (KinTek) configured for RFQ by the manufacturer, maintained at the desired temperature using a refrigerated chiller. Prepared solutions of CusF and CusB NT, 50 mM HEPES, and 5% ethylene glycol, at final mixture protein concentrations of 250 μM, were loaded anaerobically onto the apparatus via gastight syringe and rapidly mixed at the desired time point. Mixtures were quenched by liquid ethane maintained at −170 °C and the resulting snow packed firmly into machined RFQ-XAS cuvettes. The cuvettes were then further frozen in liquid nitrogen as a glassed snow for EXAFS experiments. The time resolution of the experiment was estimated to be 13 ms.

### EXAFS data collection

Samples were measured at 10 K. Cu K-edge (8.9 keV), Se K-edge (12.58 keV) extended x-ray absorption fine structure (EXAFS) and x-ray absorption near edge (XANES) data were collected at the Stanford Synchrotron Radiation Lightsource operating at 3 GeV, with currents near 500 mA maintained by continuous top off. All data were measured on beamlines 7-3 and 9-3, using a Si[220] monochromator. All data were collected in fluorescence mode using either a high-count rate Canberra 30-element (beam line 7.3) or 100-element Ge array detector (beam line 9.3) with maximal count rates per array element of <120 kHz. For each edge, 4−6 scans of buffer blank were averaged and subtracted for all protein samples in order to remove the Z-1 filter Kβ fluorescence and produce a flat pre-edge baseline. Due to the nature of the freeze quench samples, scans were taken for at least two spots on the surface of the mylar window to be assured of a homogenous sample scan and lack of ice glitches before choosing the best point to collect data. At each metal edge a Cu or Se foil, respectively, was placed between the first and second ionization chamber in order to provide energy calibration. Cu K-edges were collected using a Rh-coated mirror upstream with a 12.5 KeV energy cutoff to reject harmonics, and a nickel oxide filter and Soller slit inserted in front of the detector in order to reduce elastic scattering relative to the Cu Kα fluorescence. Se K-edges were collected using a Rh-coated mirror upstream with a 15 KeV energy cutoff to reject harmonics, and an arsenic oxide filter and Soller slit inserted in front of the detector in order to reduce elastic scattering relative to the Se Kα fluorescence.

### EXAFS simulations

Data averaging, background subtraction, and normalization to the smoothly varying background atomic absorption were performed using EXAFSPAK^48^. The experimental energy threshold energy (*k* = 0) was set to 8985 eV Cu K-edge spectra, and 12,663 eV for Se K-edge spectra. Spectral simulation was carried by least-squares curve fitting, utilizing full curved wave calculations as formulated by the SRS library program EXCURVE 9.2 as previously described^18,31,49,50^. We refined the parameters of the fit as follows: *E*_0_, the photoelectron energy threshold, *R*_*i*_ the distance of atom *i* from the central metal atom (Cu, Se) and 2σ^2^_i_ the Debye-Waller (DW) term for atom *i*. We fixed the coordination numbers to those previously established from crystal structures whenever possible, or by other previously published values as in the case of CusB NT. Determination of residual metal bound to Se in transfer experiments was performed by refining the Se−Cu shell occupancy, using a DW factor determined from simulation of the EXAFS data from a fully metallated sample. The quality of the fits was determined using the least-squares fitting parameter, *F*, which is defined as:

$$F^2 = \left( {1/N} \right)\mathop {\sum }\limits_{i = 0}^N k^6\left( {\chi _i^{{\mathrm {theory}}} - \chi _i^{{\mathrm {exp}}}} \right)^2$$

and is hereafter referred to as the fit index (FI).

### Kinetic date analysis

RFQ data generated at 23 °C were fit to a two-step kinetic mechanism involving a rapid protein−protein association followed by unimolecular metal transfer within this complex as follows

$${\mathrm {apoSeMCusF}} + {\mathrm {Cu}}\left({\mathrm I} \right){\mathrm {CusB}} \to {\mathrm {SeMCusFCu}}\left( {\mathrm I} \right){\mathrm {CusB}} \ldots k_{{\mathrm {fast}}},$$

$${\mathrm {SeMCusFCu(I)CusB}} \to {\mathrm {SeMCusFCu(I) + apoCusB}} \ldots k_{{\mathrm {slow}}}.$$

Best values of the fast second-order and slow first-order rate constants were obtained by numerical analysis using the program DYNAFIT^51^.

## Electronic supplementary material

Supplementary Information

## Data Availability

The datasets generated during and/or analyzed during the current study are available from the corresponding authors on reasonable request.
