# Supplementary material for: Trapping intermediates in metal transfer reactions of the CusCBAF export pump of Escherichia coli
Source: Commun Biol. 2018 Nov 14;1:192. doi: 10.1038/s42003-018-0181-9 (PMC6235853; doi:10.1038/s42003-018-0181-9)
Supplement: Supplementary file 1 — Supplementary Information [file 42003_2018_181_MOESM1_ESM.pdf]

<u>Supplementary information for</u>:

Trapping Intermediates in Metal Transfer Reactions of the CusCBAF Export Pump of *E. coli*

Kelly N. Chacón‡*, Jonathan Perkins‡, Zachary Mathe‡, Katherine Alwan†, Ethan N. Ho‡, Melek Nihan Ucisik§, Kenneth M. Merz‖, and Ninian J. Blackburn†*

‡Department of Chemistry and Biochemistry, Reed College, Portland, OR 97202

†Department of Biochemistry and Molecular Biology, School of Medicine, Oregon Health & Science University, Portland, OR 97239

§ Center for Drug Discovery, Baylor College of Medicine, Houston, TX 77030

‖ Department of Chemistry, Michigan State University, East Lansing, MI 48824

Supplementary Figure 1. Fourier transforms and EXAFS (insets) for WT CusF (bottom) and its M8M59I double Met to Ile mutant (top). The Figure shows the increase in the second shell amplitude due to the Se-Cu interaction resulting from mutation of non-coordinating methionine residues to Ile. Black traces are experimental data, red traces are simulated data with parameters listed in Table 1.

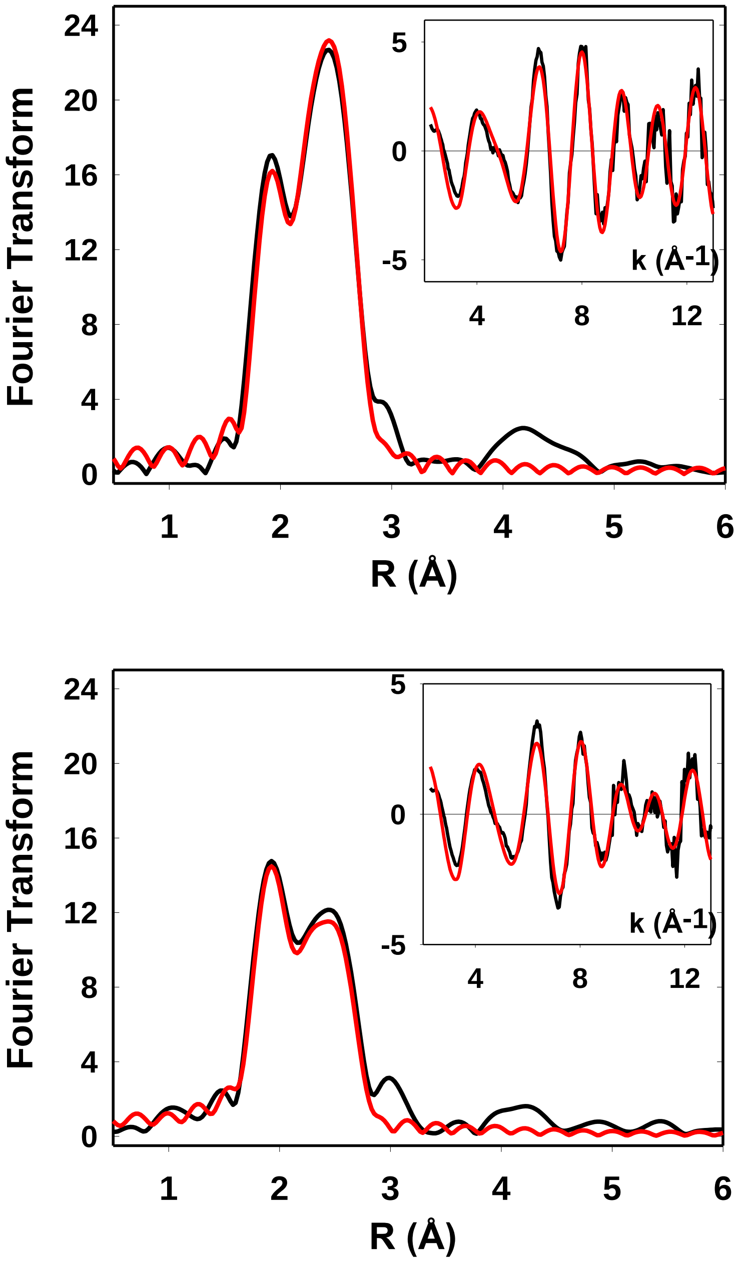

Supplementary Figure 2. The effect on Cu(I) coordination of swapping the SeM label from CusB to CusF during the transfer reaction. The figure shows Cu K edge Fourier transforms and EXAFS (insets) for the transfer intermediate formed at 500 ms between CusB and CusF. The top spectrum is the reaction between apo-SeM-CusB and Cu(I)-loaded CusF, while the bottom spectrum is the reaction between apo-SeM-CusF and Cu(I)-loaded CusB. Blue traces are experimental data and red traces are simulated with parameters given in Table 1.

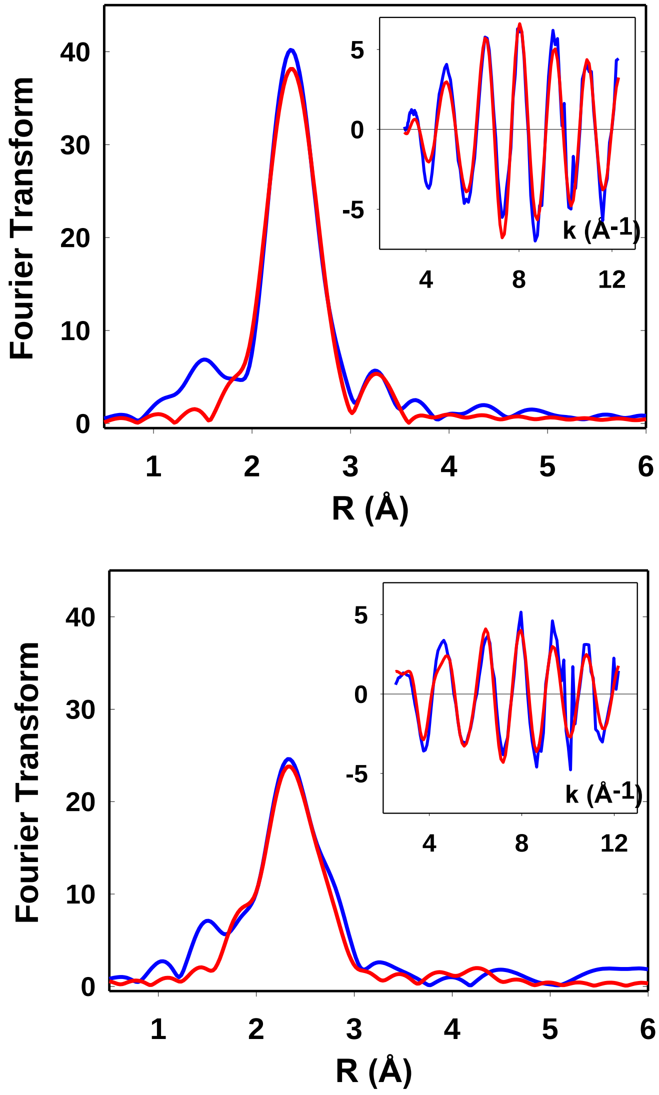

Supplementary Figure 3. Luminescence properties of apo- and Cu(I)-loaded CusF compared to its W44A variant. The variant shows no emission with or without added Cu(I). (a) Fluorescent emission with excitation at 295 nm; (b) expanded view of (a); (c) excitation profile of the 490 nm emission, showing excitation corresponding to canonical tryptophan fluorescence; (d) O_2 quenching of 490 nm emission; (e) titration of apo-CusF with Cu(I) showing decrease in 349 nm emission and concomittent increase in 486 nm emission with an end-point corresponding to 1 Cu(I) bound per protein.

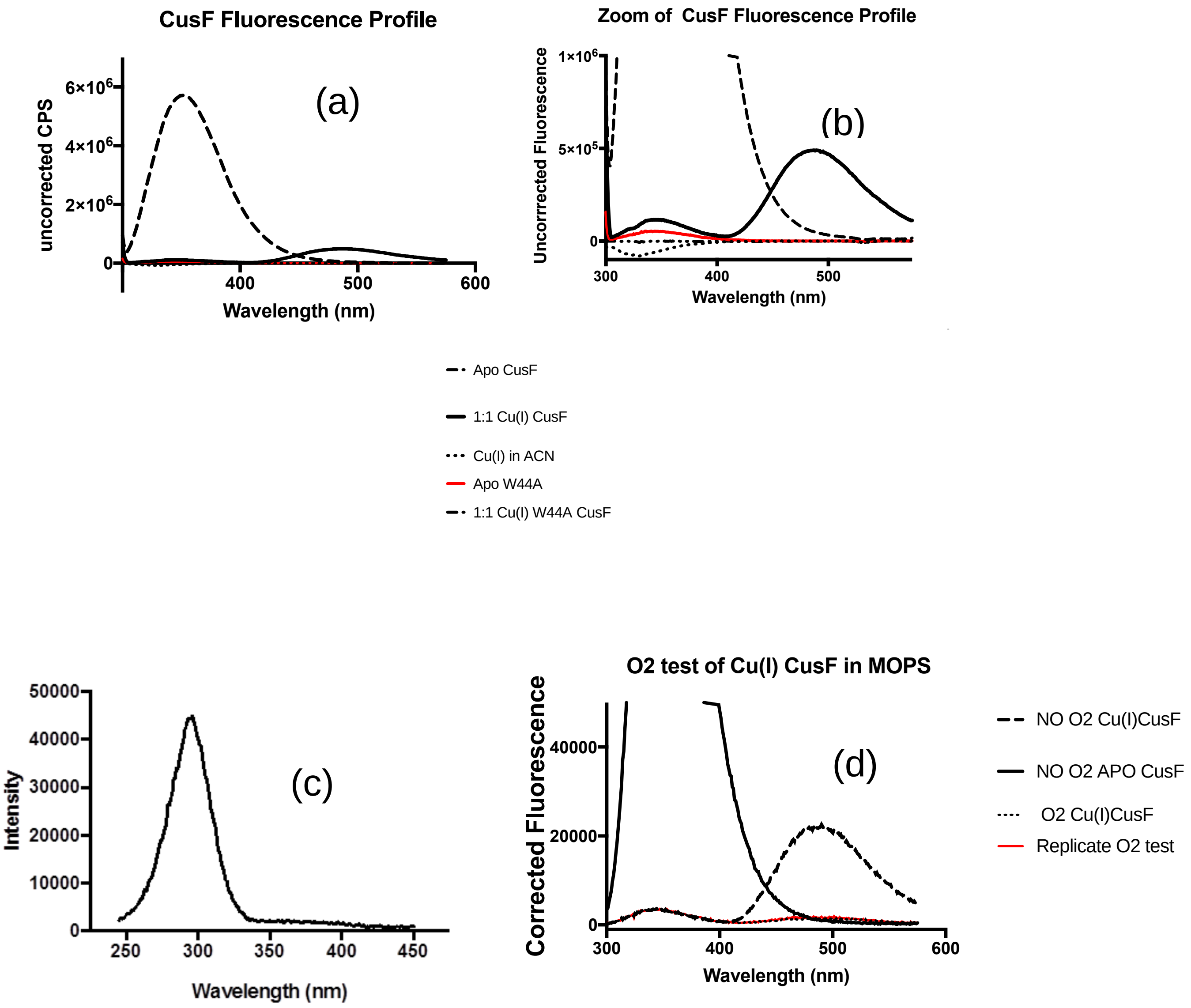

Supplementary Figure 3 (continued)

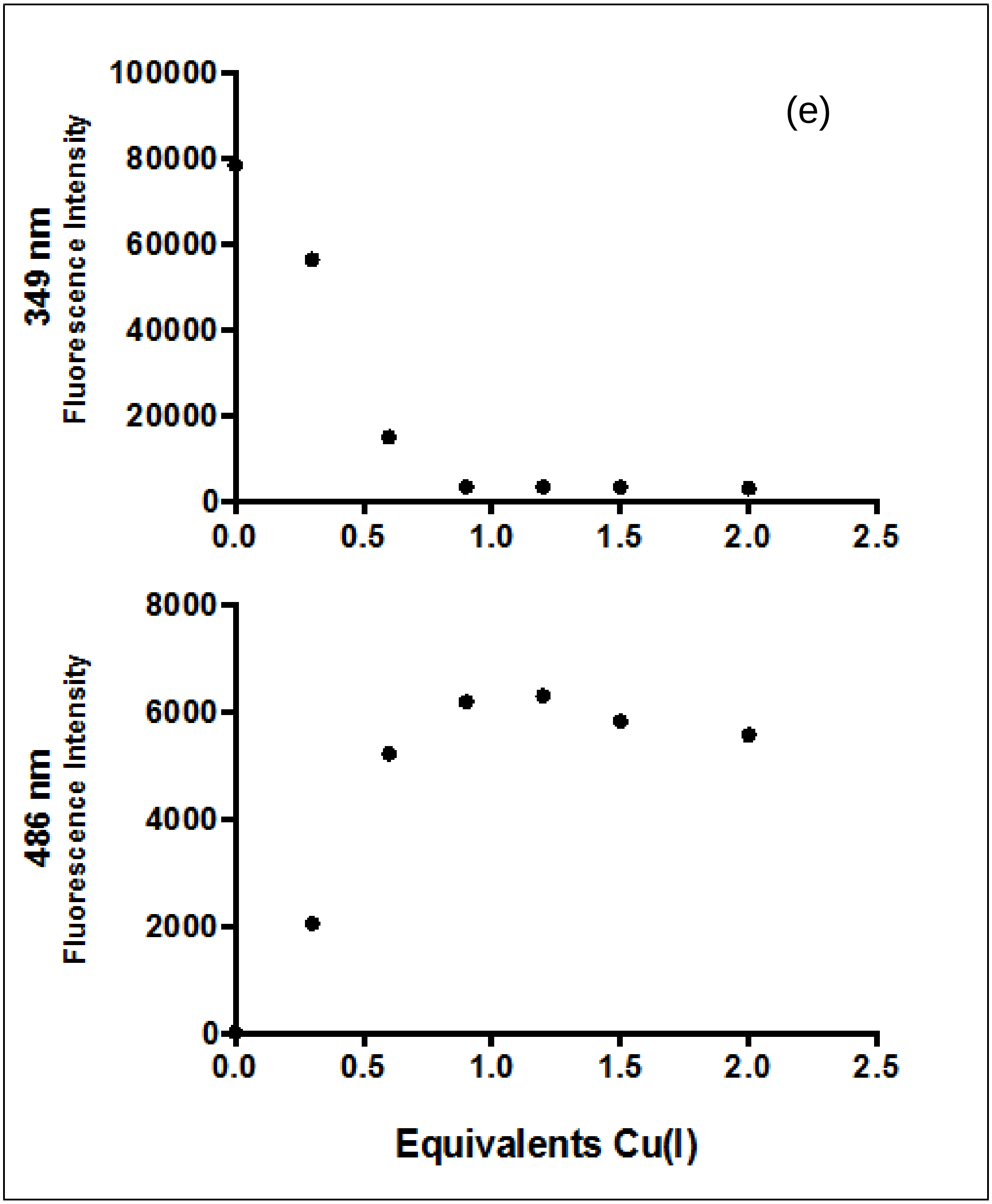

Supplementary Figure 4. Stopped flow fluorescent decay of apo-SeM-CusF reacted with Cu(I)-S(Met)-CusB-NT. The data are fitted to the same equation as for the non-labeled reaction (see Methods) and give \rate constants $k_{fast} = 98 \pm 5\ s^{-1}$ and $k_{slow} = 0.015\ s^{-1}$. The data establish that Se labeling is minimally perturbing to the rate of fluorescent decay.

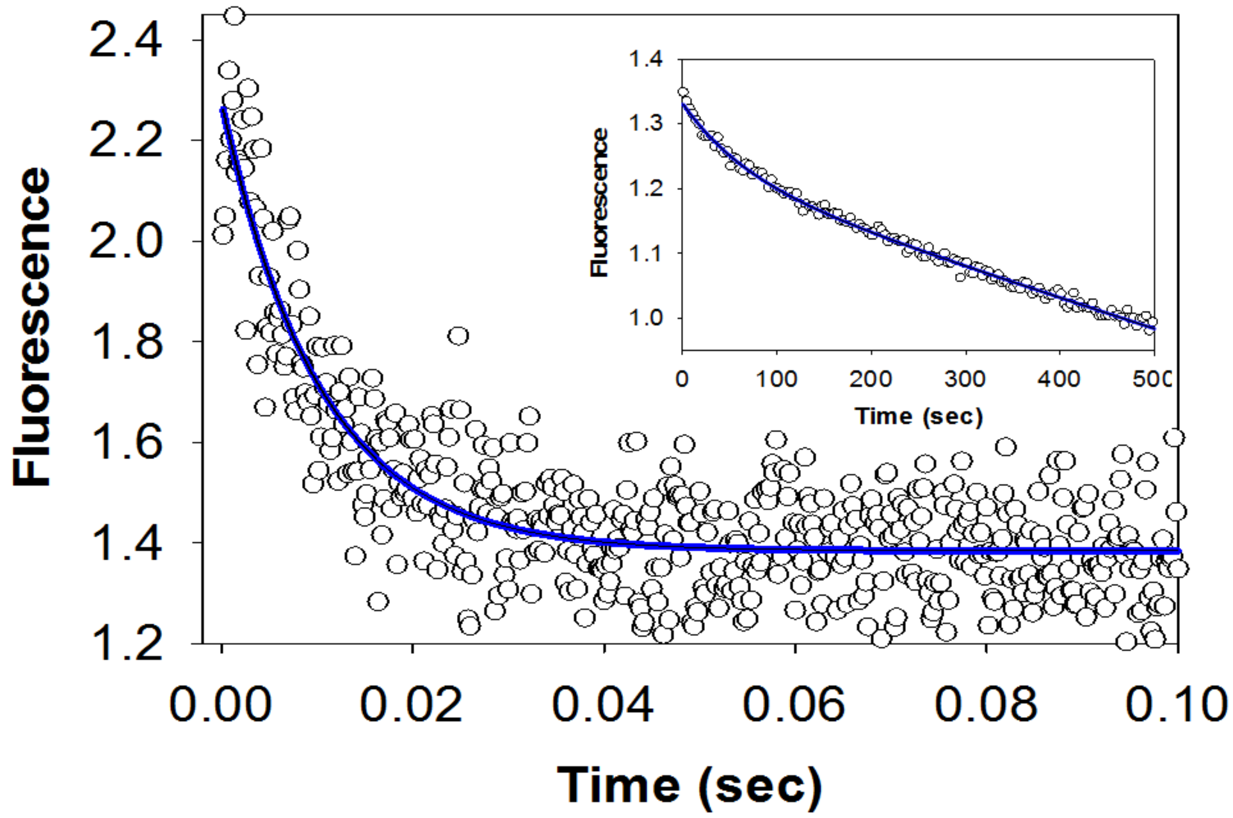

Supplementary Table 1. Shell occupation numbers for the Se-Cu interaction in RFQ generated CusF-SeMCusB mixtures at time points between 13ms and 10s. Debye Waller factors for the Se-Cu shell are fixed at $2\sigma^2$ values of 0.006 $Å^2$ for data collected at 23° C and 0.008 $Å^2$ for data collected at 4°C.

Time (ms)	S(Met)-CusB + SeMCusF 23° C		S(Met)-CusB + SeMCusF 4 ° C		SeM-CusB + S(Met)CusF 4° C	
	Shell[1] occupancy	Se-Cu Distance	Shell[1] occupancy	Se-Cu Distance	Shell[1] occupancy	Se-Cu Distance
**13**					0.54	2.41
**15**	0.60	2.41				
**26**			0.50	2.44	0.65	2.42
**46**	0.67	2.41				
**50**			0.39	2.43	0.52	2.44
**100**			0.61	2.43	0.55	2.44
**114**	0.67	2.41				
**216**	0.72	2.41				
**250**			0.52	2.44	0.66	2.44
**500**			0.46	2.46	0.53	2.44
**1000**					0.72	2.43
**1340**	0.73	2.40				
**2340**	0.75	2.40				
**3340**	0.86	2.39				
**10000**					0.62	2.43
**30000**	1.02	2.38				
